# Inhibition of neuraminidase by *Ganoderma* triterpenoids and implications for neuraminidase inhibitor design

**DOI:** 10.1038/srep13194

**Published:** 2015-08-26

**Authors:** Qinchang Zhu, Tran Hai Bang, Koichiro Ohnuki, Takashi Sawai, Ken Sawai, Kuniyoshi Shimizu

**Affiliations:** 1 Department of Agro-environmental Sciences, Faculty of Agriculture, Kyushu University, 6-10-1 Hakozaki, Higashi-ku, Fukuoka 812-8581, Japan; 2 Department of Biological and Environmental Chemistry, Kinki University, Kayanomori 11-6, Iizuka, Fukuoka 820-8555, Japan; 3 Toyotanshien Co Ltd. 3-1 Kitanijyonishi, Chuo-ku, Sapporo 060-0002, Japan

## Abstract

Neuraminidase (NA) inhibitors are the dominant antiviral drugs for treating influenza in the clinic. Increasing prevalence of drug resistance makes the discovery of new NA inhibitors a high priority. Thirty-one triterpenoids from the medicinal mushroom *Ganoderma lingzhi* were analyzed in an *in vitro* NA inhibition assay, leading to the discovery of ganoderic acid T-Q and TR as two inhibitors of H5N1 and H1N1 NAs. Structure-activity relationship studies revealed that the corresponding triterpenoid structure is a potential scaffold for the design of NA inhibitors. Using these triterpenoids as probes we found, through further *in silico* docking and interaction analysis, that interactions with the amino-acid residues Arg292 and/or Glu119 of NA are critical for the inhibition of H5N1 and H1N1. These findings should prove valuable for the design and development of NA inhibitors.

Influenza virus infection remains one of the most serious threats to human health with the potential to cause epidemics or pandemics with mass casualties. Seasonal influenza vaccines and several anti-influenza drugs are available and generally effective. However, appearance of new influenza viruses, including resistant strains, because of frequent viral antigenic drift or shift sometimes limits the effectiveness of available drugs or vaccines^1^,^2^,^3^. The two classes of antiviral drugs approved so far to treat influenza virus infection are influenza M2 ion channel blockers and neuraminidase (NA) inhibitors^4^,^5^. Because many strains of influenza virus, including the seasonal H3N2, 2009 pandemic H1N1, avian H5N1, and emerging H7N9, are now resistant to the M2 ion channel blockers amantadine (Symmetrel) and rimantadine (Flumadine), M2 ion channel blockers are now seldom used in the clinic^2^,^6^,^7^,^8^. Thus, NA inhibitors such as oseltamivir (Tamiflu) and zanamivir (Relenza) are the current standard of care for most influenza virus infections. NA cleaves glycosidic linkages to release progeny virions from infected host cells, making this enzyme crucial for the spread of influenza infection. The active site of NA is highly conserved among different influenza A subtypes and influenza B viruses^9^,^10^, so is an ideal target for the development of anti-influenza drugs. Two relatively new anti-influenza drugs, laninamivir and peramivir, are also NA inhibitors^11^.

However, drug resistance remains a challenging issue with existing NA inhibitors. Influenza A (H1N1)pdm09, which caused the most recent pandemic in 2009 and since then has circulated as a predominant seasonal strain, has now partially developed resistance to oseltamivir through the mutation of H275Y or N295S in NA^12^,^13^. In several clinical cases, oseltamivir failed to treat highly pathogenic H5N1 avian influenza because of drug resistance^14^,^15^. Therefore, there is an urgent and continuing need for new NA inhibitors.

Natural products have long been valuable sources of new drugs^16^. Their use has clear advantages over synthetic chemistry approaches in providing novel structures. In recent years, computational methodologies have become increasingly important in the drug discovery process, from hit identification and lead optimization to drug design^17^,^18^. Besides saving cost and time, a less quantifiable benefit of computer-aided drug design is the deep insight that researchers using it can gain about drug-target interactions^19^. Application of a computer-aided approach in natural product research might provide new opportunities for the discovery of NA inhibitors.

*Ganoderma lingzhi* (previously known as *Ganoderma lucidum*^20^,^21^), an oriental fungus, has been used to promote health and treat various diseases in East Asian countries, especially China, Japan, and Korea, for thousands of years^22^,^23^.The lanostane-type triterpenoids are the major bioactive constituents of *G*. *lingzhi*, and are reported to have numerous physiological activities including anti-cancer, immunomodulatory, anti-hypertensive, anti-androgenic, anti-diabetic, and antiviral properties^22^,^24^,^25^,^26^,^27^. Several triterpenoids isolated from *G. lingzhi*, such as ganoderiol F and ganoderic acid B, showed an antiviral effect against HIV-1 through inhibition of the HIV-1 protease^23^,^28^. There have been no reported anti-influenza effects of triterpenoids isolated from *G. lingzhi*. However, three triterpenoids isolated from *G. pfeifferi*, a species related to *G. lingzhi*, showed antiviral activity against influenza A^29^. They were ganodermadiol, lucidadiol and applanoxidic acid G, which showed inhibitory activity on influenza A H1N1 (A/WSN/33) in a cellular assay with an IC_50_ of greater 220 μM, 220 μM and 190 μM, respectively^29^. This study suggesting that lanostane triterpenoids from *G. lingzhi* might also have anti-influenza potential. Moreover, the triterpenoids from *G. lingzhi* have complex, highly oxidized chemical structures, similar to those of *G. pfeifferi*, further- suggesting their possible usefulness as molecular probes for activity-related sites of the NA target protein. Bioavailability is always an important issue for bioactive compounds. Although the bioavailability of *Ganoderma* triterpenoids has seldom been studied, a recent report showed that the absolute bioavailability of ganoderic acid A in rats ranged from 10.38 ~ 17.97%^30^.

Therefore, to discover potential lead compounds from *G. lingzhi* and collect structural information to guide the design of NA inhibitors, we studied 31 triterpenoids isolated from G. *lingzhi* using an NA inhibition assay and *in silico* docking, employing five NA subtypes. We compared the compounds with respect to NA inhibition, cytotoxicity, structure-activity relationships (SAR), and mode of NA binding.

## Results and Discussion

### Inhibitory activity of *Ganoderma* triterpenoids against different NA subtypes

The NA inhibition profile of *Ganoderma* triterpenoids was investigated using an *in vitro* NA inhibition assay. A total of 31 triterpenoids isolated from *G. lingzhi* were analyzed for inhibition of five NA subtypes, originating from five representative influenza strains (Table 1). NA (H1N1) was the recombinant neuraminidase originated from the 2009 pandemic influenza A (H1N1), which is also one of the current seasonal strains circulating worldwide^31^. NA (H1N1, N295S) was derived from a mutant H1N1 strain with an oseltamivir-resistant mutation, N295S, in the NA. Influenza A (H3N2) is the most prevalent seasonal strain in recent years^31^. NA (H3N2, E119V) was from a mutant H3N2 strain with the E11V mutation, also resistant to oseltamivir. NA (H5N1) was from the highly pathogenic avian influenza H5N1, while NA (H7N9) was from the emerging avian influenza H7N9^32^,^33^.

The results showed that, at 200 μM, these *Ganoderma* triterpenoids inhibited the activity of different NA subtypes to varying degrees (Table 1). For each NA subtype except NA (H7N9), ganoderic acid T-Q (1) and ganoderic acid TR (2) showed the highest levels of inhibition of all the triterpenoids. The effects of these two compounds ranged from 55.4% to 96.5% inhibition for different NA subtypes. It is interesting that most of *Ganoderma* triterpenoids showed more inhibition against N1 (neuraminidase type 1) particularly NA (H5N1) than against N2 or N9 (N1 vs. N2 or N9, P < 0.01, Wilcoxon signed rank test). For the oseltamivir-resistant enzyme NA (H1N1, N295S), ganoderic acid T-Q (1) and ganoderic acid TR (2) showed less inhibition than towards NA from the non-resistant strains, but nonetheless inhibition was 50% or greater. The lowest inhibition rates for these compounds were observed against NA (H7N9), possibly because of low amino acid sequence homology (45%) between NA (H7N9) and NA (H5N1) or NA (H1N1). These results suggest that the triterpenoids isolated from *G. lingzhi* have potential antiviral activity against N1 type influenza A, especially the H5N1 strain.

### Critical structural determinants responsible for N1 NAs inhibition by *Ganoderma* triterpenoids

Inhibition assays were performed to determine IC_50_ values of the triterpenoids against H1N1 and H5N1 NAs. As showed in Table 2, ganoderic acid T-Q (1) showed an IC_50_ of 5.6 ± 1.9 and 1.2 ± 1.0 μM against H1N1 NA and H5N1 NA, respectively. The corresponding IC_50_ values for ganoderic acid TR (2) were 4.6 ± 1.7 and 10.9 ± 6.4 μM. In addition, ganoderic acid T-N (3), ganodermanondiol (28), and lucialdehyde B (31) also showed low IC_50_ values against H5N1 NA (Table 2).

**Figure 1:**
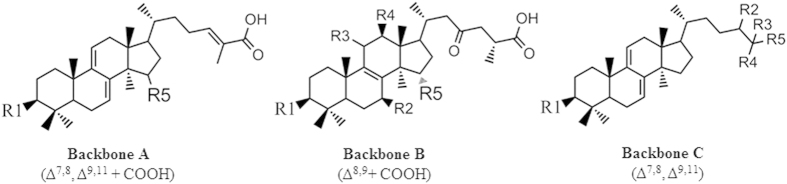
Backbone structures of the *Ganoderma* triterpenoids used in this study. Backbone A contains two double bonds (Δ^7^,^8^, Δ^9^,^11^) in the tetracyclic ring and a branch with carboxylic group. Backbone B has one double bond (Δ^8^,^9^) and a branch with carboxylic group. Backbone C has the same double bond (Δ^7^,^8^, Δ^9^,^11^) in the tetracyclic ring as backbone A but has no carboxylic group on the branch.

To identify critical structural determinates responsible for inhibition, we examined SAR of these ganoderma triterpenoids against H1N1 and H5N1 NAs. The five most active triterpenoids against NA (H1N1) and three of the five most active triterpenoids against NA (H5N1) were structures with backbone A (Table 2 and Fig. 1). This indicates that backbone A, with two double bonds in the tetracyclic ring and a branch with a carboxylic group, is a critical structural determinant for the N1 NA inhibitory activity. Further analysis of the group with backbone A indicated that triterpenoids with an acetyl or hydroxyl group at the R5 site had lower IC_50_ values than those with a hydrogen group at the R5 site. This suggests that an oxygen-containing group at the R5 site of backbone A is critical for the N1 NA inhibitory activity of *Ganoderma* triterpenoids.

### Cytotoxicity and cellular NA inhibition by *Ganoderma* triterpenoids

We assessed cytotoxicity of compounds against MCF7 cells, determining the concentration giving 50% cytotoxicity (CC_50_). Ganoderic acid T-Q (1) and ganoderic acid TR (2) had CC_50_ values of 28.2 ± 0.8 and 91.6 ± 3.4 μM, respectively (Table 2). The triterpenoids with backbone A or backbone C were generally more cytotoxic than those with backbone B, corresponding to some degree to their relative inhibitory effects against H5N1 NA. However, ganoderol B (26) inhibited H5N1 NA but showed no detectable cytotoxicity (IC_50_ = 35.5 μM and CC_50_ = >200 μM). This indicates that it should be possible to reduce the cytotoxicity of ganoderic acid T-Q and TR, while retaining NA inhibitory activity, through appropriate chemical modification.

To test the antiviral effects of ganoderic acid T-Q and TR against live influenza viruses, an MDCK cell-based assay was performed as described previously^34^ infecting with several influenza virus strains. However, because of cytotoxicity, only a weak antiviral effect could be detected with ganoderic acid TR against the oseltamivir-resistant 2009 pandemic influenza A (H1N1) virus and influenza B, with Selective Index (SI) values of 1.22 and 1.8, respectively (Supplementary Table S1). This suggests that cytotoxicity must be carefully considered in the design of NA inhibitors related to these structures.

### Binding site and interactions of *Ganoderma* triterpenoids with NA

*In silico* docking of small molecules into the structures of macromolecular targets and scoring their complementarity to the binding sites is a technique commonly used in hit identification and lead optimization. We used such docking analysis to predict the most likely binding sites and binding affinities of *Ganoderma* triterpenoids to H1N1 or H5N1 NA and to determine amino-acid residues involved in NA-triterpenoid interactions.

**Figure 2:**
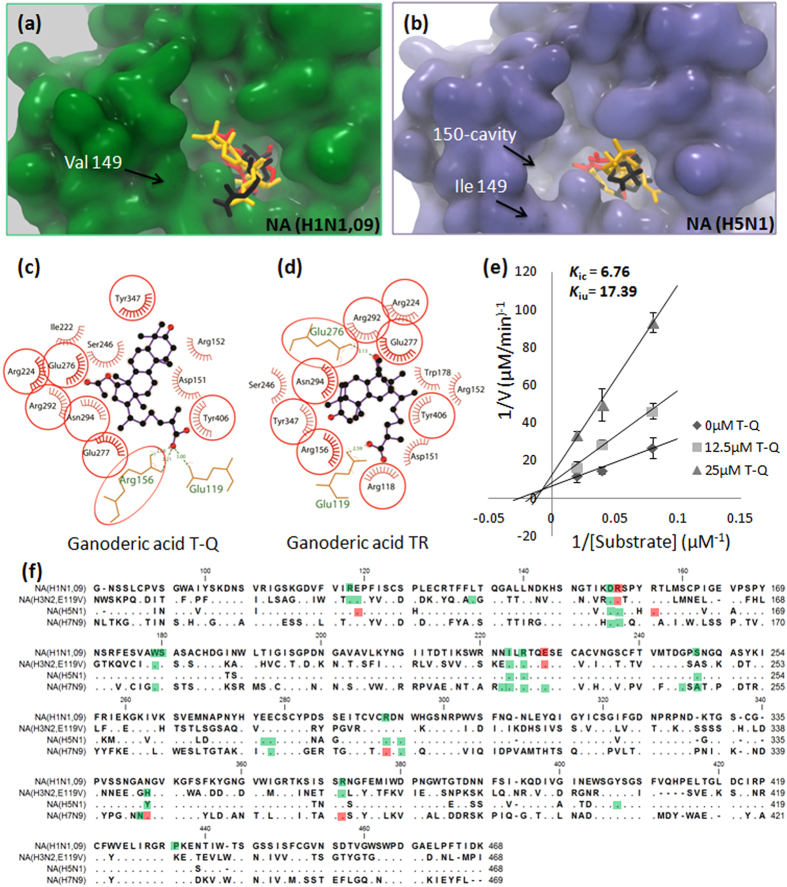
Interaction assay between NA and active *Ganoderma* triterpenoids. (**a**) Molecular surface of NA (09H1N1, PDB ID: 3TI6) with bound oseltamivir (red), ganoderic acid T-Q (yellow), or ganoderic acid TR (black). (**b**) Molecular surface of NA (H5N1, PDB ID: 2HU0) with bound oseltamivir (red), ganoderic acid T-Q (yellow), or ganoderic acid TR (black). Ligplots showing key hydrophobic and hydrogen-bonding contacts between NA (H5N1, PDB ID: 2HU0) and ganoderic T-Q (**c**) or ganoderic acid TR (**d**). The red circles indicate the same residues involved in interaction of NA with oseltamivir. (**e**) Lineweaver-Burk plot of inhibition of NA (H5N1) by ganoderic acid T-Q. *K*_ic_ and *K*_iu_ are the EI and ESI dissociation constants, respectively. (**f**) Sequence alignment between the NA subtypes and the residues in the binding pocket of NA to ganoderic acid T-Q. Matching residues were showed as dots and the gaps were showed as dashes. The red color indicates the H-bond residues, while the green color indicates the hydrophobic residues.

Oseltamivir is not only a clinically used NA inhibitor, but is also a well-known example of the successful application of computer-aided drug design. It has direct contact with eight highly conserved amino-acid residues in the active site of NA^35^. Our docking results showed that ganoderic T-Q (yellow) and TR (black) have similar binding sites as that of oseltamivir (red) (Fig. 2a,b). In contrast to the H5N1 NA and other group-1 NAs, the 2009 pandemic H1N1 NA lacks an additional 150-cavity in the active site^36^. Both ganoderic acid T-Q and TR bind to the conserved cavity of the active site but not to the non-conserved 150-cavity, an observation consistent with their broad-spectrum NA-inhibitory activity, as shown in Table 1. Upon binding, ganoderic acid T-Q was oriented in the H5N1 NA active site through hydrogen bonding of the branch carboxylic group carbonyl oxygen to Glu119 and Arg156 and hydrophobic interactions with 11 other residues (Fig. 2c). Ganoderic acid TR interacted with H5N1 NA through hydrogen bonding of the R5 hydroxyl oxygen to Glu276 and the branch carbonyl oxygen to Glu119 as well as through hydrophobic interactions with 12 other residues (Fig. 2d). Eight out of nine residues involved in the interaction between oseltamivir and H5N1 NA were also involved in the interaction between ganoderic acid T-Q and H5N1 NA. All nine of these residues were involved in the interaction between ganoderic acid TR and H5N1 NA. Thus, ganoderic acid TR had a more negative docking score, indicating a stronger binding, than ganoderic acid T-Q (Table 3). The multiple sequence alignment showed that the homology between NA (H1N1, 09) and NA (H5N1) was around 91%, while between N1 and N2 or N9 was around 45% (Fig. 2f). The residues of binding pocket for ganoderic acid T-Q indicated two conserved binding regions for this type of triterpenoids. They are region Asp151-Arg152 and region Ile222-Glu227.

Enzyme kinetics analysis revealed that the mode of inhibition of the representative active triterpenoid (ganoderic acid T-Q) on H5N1 NA is a mixed inhibition mode (Fig. 2e). That is, ganoderic acid T-Q can bind to the NA at the same time as the NA substrate and inhibit substrate binding through a possible allosteric effect. Ganoderic acid T-Q had a 2.5-fold higher affinity to free NA (*K*_*ic*_ = 6.76) than to the NA-substrate complex (*K*_*iu*_ = 17.39).

Using *in silico* docking, we compared the NA substrate, the positive control NA inhibitor, active compounds, and inactive compounds with respect to the amino-acid residues involved in the interaction with H5N1 NA or H1N1 NA (Table 3). We found that Arg292 and Glu119 are potential key residues for activity of H5N1 and H1N1 NA, particularly the H5N1 NA. They interacted with the substrate, positive control, and active compounds but not with the inactive compounds. Though Arg292 was known to be one of three conserved arginine residues in the active sites of NAs and Glu119 has been reported to be conserved among NA subtypes^37^, this is the first study to indicate that molecules interacting with NA Arg292 and/or Glu119 could inhibit binding of the natural substrate to NA.

## Conclusion

Through an *in vitro* NA inhibition assay and *in silico* analysis, this study resulted in the discovery of ganoderic acid T-Q and TR as two potential broad-spectrum inhibitors against influenza NAs, particularly H5N1 and H1N1 NAs, from the most widely known medicinal mushroom in Asia. Though cytotoxicity is an issue for them, the detailed SAR analysis performed here has indicated that it is possible to reduce their cytotoxicity by further chemical modification. In addition, taking advantage of the molecular probe function of the diversiform structures of the triterpenoids from *G. lingzhi*, this study has indicated that interaction with the amino-acid residues Arg292 and/or Glu119 of NA is critical for the inhibition of H5N1 and H1N1 NAs. Our data identifying the active triterpenoid scaffold and the key amino acid residues of NA have potentially valuable implications for the design and development of NA inhibitors.

## Methods

### Compounds and NA subtypes

Triterpenoids isolated from *G. lingzhi* were purchased from two commercial companies. Compounds 1, 3–7, 10, 14 and 21–31 (purity ≥ 97%) were purchased from Chemfaces (Wuhan, Hubei, China). Compounds 2, 8, 9, 11–13 and 15–20 (purity = 98%) were purchased from the Quality Phytochemicals, LLC (East Brunswick, NJ, USA). All compounds were dissolved in DMSO. Recombinant NAs from five Influenza A virus subtypes were purchased from a commercial source (Sino Biology Inc., Beijing, China). They are NA (A/California/04/2009/(H1N1)), NA (A/California/04/2009(H1N1)(N295S)), NA (A/Babol/36/2005(H3N2)(E119V)), NA (A/Hubei/1/2011(H5N1) and NA (A/Hangzhou/1/2013(H7N9)).

### NA inhibition assay

A modified NA inhibition assay was performed according to the manufacturer’s instructions with the NA-Fluor Influenza Neuraminidase Assay Kit (Life Technologies, Carlsbad, CA, USA). Briefly, in a well of a black 96-well plate, 1 μL sample in DMSO was mixed with 24 μL 1x assay buffer and 25 μL 300 ng/mL NA and then incubated at 37 °C for 20 min with shaking. Fluorogenic substrate was then added (50 μL of 50 μM 4-methylumbelliferyl-*N*-acetylneuraminic acid (4MUNANA)) and the plate was incubated at 37 °C for 60 min with shaking. The reaction was then terminated by adding 100 μL 0.2 M Na_2_CO_3_. Fluorescence was measured with an excitation wavelength of 355 nm and an emission wavelength of 460 nm. Relative fluorescence units (RFU) were obtained by subtracting the background value and the inhibition rate (IR) was calculated using the following formula: IR (%) = (1-RFU_sample_ /RFU_DMSO_) × 100. The difference of inhibition rate between the NA subtypes was determined by the paired Wilcoxon signed-rank test in SPSS 13.0 (SPSS, Inc., Chicago, USA). The half maximal inhibitory concentration (IC_50_) was calculated using probit regression in SPSS 13.0.

### Cytotoxicity assay

The cytotoxicity of *Ganoderma* triterpenoids against a breast cancer cell line MCF7 (Riken Cell Bank, Ibaraki, Japan) was measured with the Cell Proliferation Reagent WST-1 (Roche, Basel, Switzerland). Briefly, confluent MCF7 cells in 96-well plates were treated with test samples for 72 h. WST-1 reagent (10 μL) was then added to each well. After 2 h incubation, the formazan dye was quantified by absorbance at 450 nm in a microplate reader (Biotek-ELX800, BioTek, Winooski, VT, USA).

### Structure-activity relationship (SAR) study

The triterpenoids were divided into three groups based on their backbone structures. Backbone A has two double bonds (Δ^7^,^8^, Δ^9^,^11^) in the tetracyclic ring and a branch with carboxylic group. Backbone B has one double bond (Δ^8^,^9^) and a branch with carboxylic group. Backbone C has the same double bond (Δ^7^,^8^, Δ^9^,^11^) in the tetracyclic ring as backbone A but has no carboxylic group on the branch. The structures of the triterpenoids and their activities against NA (H1N1) or NA (H5N1) were compared.

### *In silico* docking and interaction analysis

The triterpenoids were docked to the crystal structures of H1N1 neuraminidase (N1, PDB code: 3TI6) and H5N1 neuraminidase (N1, PDB code: 2HU0) retrieved from the Protein Data Bank (PDB, www.rcsb.org), using CLC Drug Discovery Workbench software (Version 1.5, CLC Bio, Boston, MA, USA). ChemDraw Ultra 8.0 (CambridgeSoft, Cambridge, MA, USA) was first used to create two-dimensional structures of the triterpenoids. Next, three-dimensional coordinates were generated by the program Balloon (http://users.abo.fi/mivainio/balloon/)^38^. The lowest energy conformation for each compound was selected for docking. To prepare the proteins, water, ligands, and subunits were removed from the original NA structure files. Before setting up the docking target, potential binding pockets were determined using the function “Find Binding Pockets”. The predicted binding pocket that includes the active site of NA was selected as the center of the binding site, which had a radius of 13 Å. The parameter for the number of interactions for each ligand was set at 100. During docking, the conformation of the ligand was set to be changed via rotation around flexible bonds, while the protein was held as a rigid structure. After docking, only the best scoring binding mode was returned for each ligand. The PLANTS_PLP_ algorithm was used for the docking score^39^. The amino acid residues interacting with the triterpenoids and binding affinities were predicted based on the docking score, hydrogen bonding and hydrophobic interactions.

To clearly show the interaction between the triterpenoids and NA, the program LigPlot^+^ v1.4.5 (EMBL-EBI, Cambridge, United Kingdom) was used to generate schematic two-dimensional representations of NA-triterpenoid complexes obtained from the docking with the CLC Drug Discovery Workbench. The multiple sequence aligment of the NA subtypes were conducted with the CLC Drug Discovery Workbench. NA sequences including NA (H1N1, PDB 3TI6), NA (H3N2, E119V, PDB 4GZP), NA (H5N1, PDB 2HU0) and NA (H7N9, PDB 4MWQ) retrieved from the PDB. According to the docking result, the involved residues for the interaction between ganoderic acid T-Q and the NAs were indicated with color. The red color indicates the H-bond residues, while the green color indicates the hydrophobic residues.

### Determination of inhibition mode

The enzyme kinetics analysis was performed in the presence of active triterpenoids to determine their mode of inhibition. Activity of NA (H5N1) with different concentrations of substrate was measured continuously in the presence of serial concentrations of ganoderic acid T-Q using the *in vitro* NA inhibition assay protocol described above. Lineweaver-Burk plots were drawn and kinetic parameters calculated using the Exploratory Enzyme Kinetics option in SigmaPlot 12.3 (Systat Software Inc., San Jose, CA, USA).

## Additional Information

**How to cite this article**: Zhu, Q. *et al*. Inhibition of neuraminidase by *Ganoderma* triterpenoids and implications for neuraminidase inhibitor design. *Sci. Rep*. **5**, 13194; doi: 10.1038/srep13194 (2015).

## Supplementary Material

Supplementary Information

## Figures and Tables

**Table 1:** The effect of *Ganoderma* triterpenoids on the activity of NAs.

Compound	Inhibition rate (%)*					
	NA(H1N1,09)	NA(H1N1, N295S)	NA(H3N2, E119V)	NA (H5N1)	NA(H7N9)	
1.Ganoderic acid T-Q	**81.7** **±** **1.7**	**62.7** **±** **1.7**	**55.4** **±** **4.1**	**94.4** **±** **0.4**	27.4 ± 1.2	
2.Ganoderic acid TR	**87.4** **±** **1.6**	**57.7** **±** **0.9**	**59.2** **±** **1.9**	**96.5** **±** **0.3**	24.0 ± 1.8	
3.Ganoderic acid T-N	**72.0** **±** **5.5**	27.5 ± 1.4	43.6 ± 1.2	**91.1** **±** **0.8**	25.6 ± 1.6	
4.Ganoderic acid Sz	60.1 ± 4.8	29.3 ± 5.1	22.5 ± 0.3	0.0	17.8 ± 4.0	
5.Ganoderic acid S	69.4 ± 2.3	**34.1** **±** **2.2**	44.9 ± 3.2	86.7 ± 0.5	31.9 ± 2.3	
6.Ganoderic acid Y	63.0 ± 2.5	16.9 ± 2.8	23.2 ± 3.4	78.2 ± 3.2	17.8 ± 1.5	
7.Ganoderic acid A	5.0 ± 5.4	15.3 ± 1.8	32.1 ± 2.7	31.4 ± 7.3	6.3 ± 2.2	
8.Ganoderenic acid A	35.3 ± 3.7	12.4 ± 1.8	29.1 ± 2.3	35.9 ± 7.7	23.3 ± 3.1	
9.Ganoderic acid C2	32.4 ± 4.5	13.7 ± 2.4	**45.4** **±** **1.1**	60.8 ± 4.3	27.5 ± 2.3	
10.Ganoderic acid AM1	28.3 ± 6.2	9.3 ± 1.6	31.3 ± 3.2	75.2 ± 2.3	14.1 ± 5.1	
11.Ganoderic acid K	28.8 ± 2.0	14.2 ± 2.9	33.5 ± 2.4	57.7 ± 10.9	28.5 ± 1.9	
12.Ganoderenic acid H	58.0 ± 3.0	12.8 ± 5.1	38.6 ± 2.3	77.7 ± 3.9	**38.6** **±** **6.2**	
13.Ganoderic acid H	35.0 ± 4.5	16.9 ± 2.4	27.9 ± 1.5	64.1 ± 4.5	27.2 ± 3.2	
14.Ganoderic acid B	6.9 ± 6.8	12.8 ± 1.4	38.7 ± 1.6	3.0 ± 1.0	5.5 ± 0.4	
15.Ganoderenic acid F	53.1 ± 1.6	24.4 ± 4.4	24.2 ± 3.9	62.8 ± 4.0	**34.2** **±** **4.7**	
16.Ganoderenic acid C	27.9 ± 0.3	13.2 ± 2.2	37.5 ± 2.4	30.6 ± 4.5	27.2 ± 0.5	
17.Ganoderenic acid D	50.4 ± 3.0	13.8 ± 3.1	32.2 ± 3.4	74.2 ± 2.2	**35.9** **±** **2.8**	
18.Ganoderic acid C6	17.8 ± 1.2	17.6 ± 3.5	20.7 ± 0.5	15.6 ± 22.3	25.7 ± 1.3	
19.Ganoderic acid C1	21.1 ± 4.1	14.7 ± 3.9	32.0 ± 3.4	43.3 ± 6.1	26.2 ± 4.1	
20.Ganoderic acid DM	50.4 ± 1.5	19.0 ± 2.4	28.8 ± 0.5	55.2 ± 0.7	29.4 ± 3.8	
21.Ganolucidic acid A	19.4 ± 2.7	21.0 ± 4.2	22.9 ± 3.8	49.8 ± 7.7	16.7 ± 3.4	
22.Ganoderic acid Zeta	35.9 ± 5.3	25.5 ± 4.9	18.7 ± 4.5	0.0	17.7 ± 2.4	
23.Ganoderic acid LM2	40.9 ± 6.2	15.4 ± 0.6	12.9 ± 1.0	67.1 ± 5.2	12.7 ± 9.6	
24.Ganoderic acid F	12.9 ± 11.4	17.6 ± 2.0	19.9 ± 2.6	48.6 ± 7.4	14.8 ± 2.6	
25.Ganoderol A	48.3 ± 2.2	22.6 ± 1.8	18.2 ± 2.7	77.1 ± 0.4	15.9 ± 3.7	
26.Ganoderol B	51.0 ± 2.6	19.9 ± 1.1	19.1 ± 6.1	71.6 ± 2.1	21.8 ± 3.5	
27.Ganoderiol F	52.9 ± 2.0	9.5 ± 4.5	16.8 ± 1.2	33.6 ± 12.1	29.5 ± 4.3	
28.Ganodermanondiol	53.1 ± 2.5	19.0 ± 4.2	34.2 ± 0.9	87.9 ± 1.8	24.3 ± 1.7	
29.Ganodermanontriol	35.3 ± 5.2	7.9 ± 3.2	12.2 ± 3.4	60.5 ± 6.8	10.3 ± 3.5	
30.Lucialdehyde A	57.6 ± 1.7	5.8 ± 2.4	25.9 ± 8.1	67.1 ± 5.7	24.5 ± 1.6	
31.Lucialdehyde B	51.4 ± 2.5	11.2 ± 1.3	43.3 ± 1.0	86.7 ± 0.8	30.4 ± 3.5	

^*^Inhibition rates were calculated from independent NA inhibition assays (n = 3) that used 200 μM of each compound and different NA subtypes. They are expressed as means ± standard deviation. The values showed in boldface are the top three inhibitors against each NA subtype. The difference between NA subtypes were determined by the paired Wilcoxon signed-rank test (n = 31): NA (H1N1, 09) vs. NA (H3N2, E119V), P = 0.002; NA (H1N1, 09) vs. NA (H7N9), P = 0.00001; NA (H5N1) vs. NA (H3N2, E119V), P = 0.00003; NA (H5N1) vs. NA (H7N9), P = 0.000008; NA (H1N1, N295S) vs. NA (H7N9), P0= 0.085; NA (H1N1, 09) vs. NA (H1N1, N295S), P = 0.000005.

**Table 2:** Results of NA inhibition assay, cytotoxicity assay, and SAR analysis.

Compound	Structure							IC_50_ (μM)^a^		CC_50_ (μM)^b^
	Backbone	R1	R2	R3	R4	R5	Bond/Abs. confg.	NA (H1N1,09)	NA (H5N1)	
1.Ganoderic acid T-Q	A	=O	—	—	—	−OCOCH3	Δ^24^,^25^(E)	5.6 ± 1.9	1.2 ± 1.0	28.2 ± 0.8
2.Ganoderic acid TR	A	=O	—	—	—	−OH	Δ^24^,^25^(E)	4.6 ± 1.7	10.9 ± 6.4	91.6 ± 3.4
3.Ganoderic acid T-N	A	−OH	—	—	—	−OCOCH3	Δ^24^,^25^(E)	42.0 ± 13.5	2.7 ± 0.4	24.4 ± 2.4
4.Ganoderic acid Sz	A	=O	—	—	—	−H	Δ^24^,^25^(Z)	100.9 ± 35.9	>200	50.6 ± 0.1
5.Ganoderic acid S	A	=O	—	—	—	−H	Δ^24^,^25^(E)	80.5 ± 24.9	>200	80.9 ± 6.8
6.Ganoderic acid Y	A	−OH	—	—	—	−H	Δ^24^,^25^(E)	>200	>200	18.0 ± 1.3
7.Ganoderic acid A	B	=O	−OH	=O	−H	−OH	C^25^(R)	>200	>200	>200
8.Ganoderenic acid A	B	=O	−−OH	=O	−H	−OH	Δ^20^,^22^(E)	>200	>200	>200
9.Ganoderic acid C2	B	−OH	−OH	=O	−H	−OH	C^25^(R)	>200	>200	>200
10.Ganoderic acid AM1	B	−OH	=O	=O	−H	=O	C^25^(R)	>200	135.3 ± 24.6	>200
11.Ganoderic acid K	B	−OH	−OH	=O	−OCOCH_3_	=O	Not defined	>200	173.0 ± 5.2	>200
12.Ganoderenic acid H	B	−OH	=O	=O	−H	=O	Δ^20^,^22^(E)	>200	28.0 ± 10.9	>200
13.Ganoderic acid H	B	−OH	=O	=O	−OCOCH_3_	=O	C^20^(S)	>200	143.9 ± 46.3	>200
14.Ganoderic acid B	B	−OH	−OH	=O	−H	=O	C^20^(S), C^25^(R)	>200	>200	>200
15.Ganoderenic acid F	B	=O	=O	=O	−H	=O	Δ^20^,^22^(E)	>200	142.6 ± 43.1	110.6 ± 17.9
16.Ganoderenic acid C	B	−OH	−OH	=O	−H	−OH	Δ^20^,^22^(E)	>200	>200	>200
17.Ganoderenic acid D^c^	B	=O	−OH	=O	−H	=O	Δ^20^,^22^(E)	>200	123.4 ± 22.5	>200
18.Ganoderic acid C6	B	−OH	=O	=O	−OH	=O	C^25^(R)	>200	>200	>200
19.Ganoderic acid C1	B	=O	−OH	=O	−H	=O	C^25^(R)	>200	>200	>200
20.Ganoderic acid DM	B	=O	=O	−H	−H	−H	Δ^24^,^25^(E)	>200	>200	>200
21.Ganolucidic acid A	B	=O	−H	=O	−H	−OH	C^25^(R)	>200	>200	>200
22.Ganoderic acid Zeta	B	−OH	=O	=O	−H	=O	C^23^(OH), Δ^24^,^25^(E)	>200	>200	>200
23.Ganoderic acid LM2^d^	B	=O	−OH	=O	−H	=O	C^23^(OH), Δ^24^,^25^(E)	>200	130.0 ± 25.5	>200
24.Ganoderic acid F	B	=O	=O	=O	−OCOCH3	=O	C^20^(R)	>200	>200	>200
										
25.Ganoderol A	C	=O	−H	—	−CH3	−CH2OH	Δ^24^,^25^(E)	>200	60.3 ± 13.7	20.4 ± 0.9
26.Ganoderol B	C	−OH	−H	—	−CH3	−CH2OH	Δ^24^,^25^(E)	>200	35.5 ± 11	>200
27.Ganoderiol F	C	=O	−H	—	−CH2OH	−CH2OH	Δ^24^,^25^	>200	>200	>200
28.Ganodermanondiol	C	=O	−OH	−OH	−CH3	−CH3	C^24^(S)	>200	2.7 ± 0.6	64.9 ± 10.0
29.Ganodermanontriol	C	=O	−OH	−OH	−CH3	−CH2OH	C^24^(S), C^25^(R)	>200	>200	>200
30.Lucialdehyde A	C	−OH	−H	—	−CH3	−CHO	Δ^24^,^25^(E)	>200	164.3 ± 18.0	34.7 ± 5.5
31.Lucialdehyde B^e^	C	=O	−H	—	−CH3	−CHO	Δ^24^,^25^(E)	>200	1.8 ± 1.6	7.1 ± 0.3

^a^IC_50_ was obtained from the *in vitro* NA inhibition assay (n = 3).

^b^CC_50_ was obtained from the cytotoxicity assay with MCF-7 cells (n = 3).

^c^Branch does not bear −C = O group at C^23^.

^d^Branch bears -OH group at C^23^.

^e^Double bonds Δ^7^,^8^ and Δ^9^,^11^ are replaced by Δ^8^,^9^, and C^7^ is changed to –C = O; (Δ): double bond; (Abs. confg.): absolute configuration; “−”: does not exist.

**Table 3:** Binding modes of substrate, active compounds, or inactive compounds to H5N1 or H1N1 NA.

	NA(H5N1)					NA(H1N1,09)				
	4MUNANA^a^	Oseltamivir	Ganoderic acid T-Q	Ganoderic acid TR	Ganoderic acid SZ^b^	4MUNANA^a^	Oseltamivir	Ganoderic acid T-Q	Ganoderic acid TR	Ganoderic acid Y^b^
**Hydrogen bonding**	Arg156	Arg156	**Glu119**	**Glu119**	Tyr347	Arg118	Arg152	Arg152	Asp151	Asp151
	**Arg292**	**Arg292**	Arg156	Glu276	Arg156	**Arg292**	Arg118	Glu227	Arg152	Asp152
	Asn294	Tyr347				Asn347	**Arg292**		Glu277	Asn347
	Tyr406					Arg371	Arg371		**Arg292**	
						Tyr406			Tyr406	
**Hydrophobic**	Arg118	Arg224	Asp151	Arg118	Asp151	Ile149	**Glu119**	Arg118	**Glu119**	Ile149
	**Glu119**	Glu276	Arg152	Asp151	Arg152	Asp151	Asp151	Asp151	Trp178	Lys150
	Asp151	Asn294	Ile222	Arg152	Trp178	Arg152	Ile222	Trp178	Ile222	
	Trp178	Asn294	Arg224	Arg156	Ile222	Ile222	Arg224	Ser179	Arg224	
	Ser179	Tyr406	Ser246	Trp178	Arg224	Arg224	Ser246	Ile222	Glu227	
	Glu227	Arg118	Glu276	Arg224	Ser246	Glu277	Glu276	Arg224	Ser246	
	Glu277	Glu277	Glu277	Ser246	Glu276		Glu277	Ser246	Glu276	
	Tyr347		**Arg292**	Glu277	Glu277		Try406	**Arg292**		
			Asn294	**Arg292**	Asn294			Arg371		
			Tyr347	Asn294	Tyr406			Pro431		
			Tyr406	Tyr347						
				Try406						
**Score**^C^	−46.3	−35.1	−33.9	−39.1	−35.6	−47.2	−40.9	−35.1	−43.3	−38.2

^a^4MUNANA is the substrate used in the NA inhibition assay; the amino acid residues in bold are common residues involved in the interaction between NA and the substrate or active compounds but not inactive compounds.

^b^Inactive compounds for NA inhibition (IC_50_ > 200 μM).

^C^Scores were obtained from the docking assay, reflecting the binding affinity between the target protein and the ligand.

## References

[b1] Medina R. A. & Garcia-Sastre A. Influenza A viruses: new research developments. Nat Rev Microbiol 9, 590–603 (2011). 21747392 10.1038/nrmicro2613 PMC10433403

[b2] Oh D. Y. & Hurt A. C. A Review of the Antiviral Susceptibility of Human and Avian Influenza Viruses over the Last Decade. Scientifica (Cairo) 2014, 430629 (2014). 24800107 10.1155/2014/430629 PMC3995103

[b3] Osterholm M. T., Kelley N. S., Sommer A. & Belongia E. A. Efficacy and effectiveness of influenza vaccines: a systematic review and meta-analysis. Lancet Infect Dis 12, 36–44 (2012). 22032844 10.1016/S1473-3099(11)70295-X

[b4] Barik S. New treatments for influenza. BMC Med 10, 104 (2012). 22973873 10.1186/1741-7015-10-104 PMC3523090

[b5] De Clercq E. Antiviral agents active against influenza A viruses. Nat Rev Drug Discov 5, 1015–1025 (2006). 17139286 10.1038/nrd2175 PMC7097821

[b6] Girard M. P., Tam J. S., Assossou O. M. & Kieny M. P. The 2009 A (H1N1) influenza virus pandemic: A review. Vaccine 28, 4895–4902 (2010). 20553769 10.1016/j.vaccine.2010.05.031

[b7] Bright R. A., Shay D. K., Shu B., Cox N. J. & Klimov A. I. Adamantane resistance among influenza A viruses isolated early during the 2005-2006 influenza season in the United States. JAMA 295, 891–894 (2006). 16456087 10.1001/jama.295.8.joc60020

[b8] He G. . Amantadine-resistance among H5N1 avian influenza viruses isolated in Northern China. Antiviral Res 77, 72–76 (2008). 17897729 10.1016/j.antiviral.2007.08.007

[b9] Colman P. M., Hoyne P. A. & Lawrence M. C. Sequence and structure alignment of paramyxovirus hemagglutinin-neuraminidase with influenza virus neuraminidase. J Virol 67, 2972–2980 (1993). 8497041 10.1128/jvi.67.6.2972-2980.1993 PMC237633

[b10] Air G. M. Influenza neuraminidase. Influenza Other Respir Viruses 6, 245–256 (2012). 22085243 10.1111/j.1750-2659.2011.00304.x PMC3290697

[b11] Chairat K., Tarning J., White N. J. & Lindegardh N. Pharmacokinetic properties of anti-influenza neuraminidase inhibitors. J Clin Pharmacol 53, 119–139 (2013). 23436258 10.1177/0091270012440280

[b12] Hurt A. C. . Antiviral resistance during the 2009 influenza A H1N1 pandemic: public health, laboratory, and clinical perspectives. Lancet Infect Dis 12, 240–248 (2012). 22186145 10.1016/S1473-3099(11)70318-8

[b13] Morlighem J. E. . Mutation analysis of 2009 pandemic influenza A(H1N1) viruses collected in Japan during the peak phase of the pandemic. PLoS One 6, e18956 (2011). 21572517 10.1371/journal.pone.0018956 PMC3084724

[b14] Le Q. M. . Avian flu: isolation of drug-resistant H5N1 virus. Nature 437, 1108 (2005). 16228009 10.1038/4371108a

[b15] de Jong M. D. . Oseltamivir resistance during treatment of influenza A (H5N1) infection. N Engl J Med 353, 2667–2672 (2005). 16371632 10.1056/NEJMoa054512

[b16] Newman D. J. & Cragg G. M. Natural products as sources of new drugs over the 30 years from 1981 to 2010. Journal of natural products 75, 311–335 (2012). 22316239 10.1021/np200906s PMC3721181

[b17] Kitchen D. B., Decornez H., Furr J. R. & Bajorath J. Docking and scoring in virtual screening for drug discovery: methods and applications. Nat Rev Drug Discov 3, 935–949 (2004). 15520816 10.1038/nrd1549

[b18] Veselovsky A. V. & Ivanov A. S. Strategy of computer-aided drug design. Curr Drug Targets Infect Disord 3, 33–40 (2003). 12570731 10.2174/1568005033342145

[b19] Kerry P. S. . Structural basis for a class of nanomolar influenza A neuraminidase inhibitors. Sci Rep 3, 2871 (2013). 24129600 10.1038/srep02871 PMC3797432

[b20] Cao Y., Wu S.-H. & Dai Y.-C. Species clarification of the prize medicinal Ganoderma mushroom “Lingzhi”. Fungal Diversity 56, 49–62 (2012).

[b21] Bishop K. S. . From 2000 years of Ganoderma lucidum to recent developments in nutraceuticals. Phytochemistry 114, 56–65 (2015). 25794896 10.1016/j.phytochem.2015.02.015

[b22] Paterson R. R. Ganoderma - a therapeutic fungal biofactory. Phytochemistry 67, 1985–2001 (2006). 16905165 10.1016/j.phytochem.2006.07.004

[b23] Wasser S. P. Reishi or Ling Zhi (Ganoderma lucidum). Encyclopedia of Dietary Supplements 1, 603–622 (2005).

[b24] Wu G. S. . Anti-cancer properties of triterpenoids isolated from Ganoderma lucidum - a review. Expert Opin Investig Drugs 22, 981–992 (2013). 10.1517/13543784.2013.805202 23790022

[b25] Liu J., Shimizu K. & Kondo R. The effects of ganoderma alcohols isolated from Ganoderma lucidum on the androgen receptor binding and the growth of LNCaP cells. Fitoterapia 81, 1067–1072 (2010). 20603196 10.1016/j.fitote.2010.06.029

[b26] Boh B., Berovic M., Zhang J. & Zhi-Bin L. Ganoderma lucidum and its pharmaceutically active compounds. Biotechnol Annu Rev 13, 265–301 (2007). 17875480 10.1016/S1387-2656(07)13010-6

[b27] Fatmawati S., Shimizu K. & Kondo R. Ganoderol B: a potent alpha-glucosidase inhibitor isolated from the fruiting body of Ganoderma lucidum. Phytomedicine 18, 1053–1055 (2011). 21596546 10.1016/j.phymed.2011.03.011

[b28] el-Mekkawy S. . Anti-HIV-1 and anti-HIV-1-protease substances from Ganoderma lucidum. Phytochemistry 49, 1651–1657 (1998). 9862140 10.1016/s0031-9422(98)00254-4

[b29] Mothana R. A. . Antiviral lanostanoid triterpenes from the fungus Ganoderma pfeifferi. Fitoterapia 74, 177–180 (2003). 12628419 10.1016/s0367-326x(02)00305-2

[b30] Jin Z. . Pharmacokinetics and Oral Bioavailability of Ganoderic Acid A by High Performance Liquid Chromatography-Tandem Mass Spectrometry. International Journal Of Pharmacology 11, 27–34 (2015).

[b31] WHO. Influenza update. http://www.who.int/influenza/surveillance_monitoring/updates/en/ (Accessed June 5, 2015).

[b32] Wang Y. . Towards a better understanding of the novel avian-origin H7N9 influenza A virus in China. Sci Rep 3, 2318 (2013). 23897131 10.1038/srep02318 PMC3727058

[b33] Chen Y. . Human infections with the emerging avian influenza A H7N9 virus from wet market poultry: clinical analysis and characterisation of viral genome. Lancet 381, 1916–1925 (2013). 23623390 10.1016/S0140-6736(13)60903-4 PMC7134567

[b34] Watanabe K., Rahmasari R., Matsunaga A., Haruyama T. & Kobayashi N. Anti-influenza viral effects of honey *in vitro*: potent high activity of manuka honey. Arch Med Res 45, 359–365 (2014). 24880005 10.1016/j.arcmed.2014.05.006

[b35] Von Itzstein M. The war against influenza: discovery and development of sialidase inhibitors. Nat Rev Drug Discov 6, 967–974 (2007). 18049471 10.1038/nrd2400

[b36] Li Q. . The 2009 pandemic H1N1 neuraminidase N1 lacks the 150-cavity in its active site. Nat Struct Mol Biol 17, 1266–1268 (2010). 20852645 10.1038/nsmb.1909

[b37] Russell R. J. . The structure of H5N1 avian influenza neuraminidase suggests new opportunities for drug design. Nature 443, 45–49 (2006). 16915235 10.1038/nature05114

[b38] Vainio M. J. & Johnson M. S. Generating conformer ensembles using a multiobjective genetic algorithm. J Chem Inf Model 47, 2462–2474 (2007). 17892278 10.1021/ci6005646

[b39] Korb O., Stutzle T. & Exner T. E. Empirical scoring functions for advanced protein-ligand docking with PLANTS. J Chem Inf Model 49, 84–96 (2009). 19125657 10.1021/ci800298z

