# Supplementary material for: Inhibition of neuraminidase by Ganoderma triterpenoids and implications for neuraminidase inhibitor design
Source: Sci Rep. 2015 Aug 26;5:13194. doi: 10.1038/srep13194 (PMC4549708; doi:10.1038/srep13194)
Supplement: Supplementary Information [file srep13194-s1.pdf]

Supplementary Information

Inhibition of neuraminidase by *Ganoderma* triterpenoids and implications for neuraminidase inhibitor design

Qinchang Zhu[1], Tran Hai Bang[1], Koichiro Ohnuki[2], Takashi Sawai[3], Ken Sawai[3], Kuniyoshi Shimizu[1, *]

Author affiliations:

1, Department of Agro-environmental Sciences, Faculty of Agriculture, Kyushu University, 6-10-1 Hakozaki, Higashi-ku, Fukuoka 812-8581, Japan

2, Department of Biological and Environmental Chemistry, Kinki University, Kayanomori 11-6, Iizuka, Fukuoka 820-8555, Japan

3, Toyotanshien Co Ltd. 3-1 Kitanijyonishi, Chuo-ku, Sapporo 060-0002, Japan

*Corresponding author. Telephone/Fax: +81-92-642-3002. E-mail address: shimizu@agr.kyushu-u.ac.jp (K. Shimizu).

Supplementary Table S1. Results of cellular anti-influenza virus assay

	Ganoderic acid T-Q			Ganoderic acid TR		
	CC50 (μM)	IC50 (μM)	SI	CC50 (μM)	IC50 (μM)	SI
H1N1 (A/HA-4pdm)	41.1	n.d.	-	298.7	n.d.	-
H1N1 (A/HA-58pdm-TR)	41.1	n.d.	-	298.7	245.4	1.22
H3N2 (A/Hong Kong/8/68)	41.1	n.d.	-	298.7	n.d.	-
H5N2 (A/Duck/Pennsylvania/84)	41.1	n.d.	-	298.7	324.3	0.92
Influenza B (B/Lee/40)	41.1	189.9	0.22	298.7	166.4	1.8

Note: Cellular anti-influenza virus assay was performed by the ANTI VIRUS SCREENING SYSTEM Corporation (AVSS, Co., Nagasaki, Japan). The H1N1 (A/HA-4pdm) is an isolated strain of the 2009 pandemic influenza A (H1N1). H1N1 (A/HA-58pdm-TR) is the oseltamivir-resistant strain of H1N1 (A/HA-4pdm). The cytopathic effect (CPE) of the influenza viruses (100 $TCID_{50}$) on MDCK cells (Madin-Darby Canine Kidney cells) was measured in the presence of different concentrations of ganoderic acid T-Q or TR. CC_{50} is the concentration to cause 50% cytotoxicity to the cells. IC_{50} is the concentration to inhibit the CPE of viruses by 50%. SI, selective index, equal to CC_{50}/ IC_{50}. n.d.: Not determined.
